# Comparison of IA‐2 Bridge ELISA and Radiobinding Assays for Progression Risk Assessment in Early‐Stage Type 1 Diabetes

**DOI:** 10.1111/dom.70630

**Published:** 2026-03-03

**Authors:** Ezio Bonifacio, Marlon Scholz, Andreas Weiss, Anette‐Gabriele Ziegler

**Affiliations:** ^1^ Paul Langerhans Institute Dresden of the Helmholtz Munich at University Hospital Carl Gustav Carus and Faculty of Medicine TU Dresden Dresden Germany; ^2^ Institute of Diabetes Research, Helmholtz Munich German Research Center for Environmental Health Munich Germany

**Keywords:** cohort study, pancreatic autoantibodies, risk prediction, type 1 diabetes

## 1 Background

Type 1 diabetes can be diagnosed in early presymptomatic stages defined by two or more islet autoantibodies without (Stage 1) and with accompanying dysglycaemia (Stage 2) [1]. Stage 2 disease is associated with a relatively rapid progression to clinical diabetes (Stage 3) [2], providing opportunities to assess the efficacy of potential disease‐modifying therapies to delay the onset of stage 3. Individuals with stage 2, however, represent a minority of those diagnosed with early‐stage disease, which can lead to long recruitment periods for clinical trials. We have developed a Progression Likelihood Score (PLS), which stratifies progression rates in children with stage 1 disease and identifies a significant number with a PLS > 4.0 in whom progression rates were similar to those seen in stage 2 disease [3, 4]. In addition to HbA1c and the 90‐min blood glucose value during the oral glucose tolerance test (OGTT), the PLS includes quantitative measurement of IA‐2 autoantibodies (IA‐2A), which are a marker for faster progression [5]. IA‐2A were measured by harmonized radiobinding assays (RBA) [6], which are not certified assays and are not readily available or used in clinical practice, thereby limiting the use of PLS for eligibility in clinical trials and for identifying individuals with stage 1 who require more frequent monitoring. Assays often used by clinical laboratories are a bridge ELISA produced by RSR limited and commercialised as the RSR IA‐2 Autoantibody ELISA version 2 or as the KRONUS IA‐2 autoantibody ELISA kit. The objective of this study was to align the thresholds required to calculate the PLS across the IA‐2A RBA and commercially available ELISA assays. Establishing a PLS that can be derived from widely available commercial assays will increase its accessibility for stratifying risk in individuals with stage 1 type 1 diabetes and enable the use of the PLS to extend enrolment of individuals with fast progression rates to stage 3 into clinical trials.

## 2 Methods

Serum samples from 349 children with stage 1 type 1 diabetes (166 girls, median age 4.4 (IQR, 3.3–5.7) years, including 54 with a first degree family history with type 1 diabetes) participating in the Bavarian Fr1da study were used for this comparison [4, 7]. Samples were taken at the same visit at which an OGTT and HbA1c assessment were performed; the 90‐min glucose during OGTT, the HbA1c, and the IA‐2A category defined by IA‐2A titre were used to calculate the PLS [3]. Stage 1 was defined as normal glucose tolerance—fasting plasma glucose < 5.6 mmol/L (100 mg/dL), 2‐h OGTT plasma glucose < 7.8 mmol/L (140 mg/dL) and OGTT plasma glucose < 11.1 mmol/L (200 mg/dL) at 30, 60 and 90 min—together with HbA1c < 39 mmol/mol (5.7%). Children were followed for the development of stage 3 (median follow‐up 3.9 years [IQR, 2.0–6.5]), which was the study outcome and was defined using the 2025 ADA criteria [8].

IA‐2A were measured by RBA and expressed as NIDDK units as previously described [6] and using the RSR IA‐2 Autoantibody ELISA version 2 assay (RSR Ltd., Cardiff UK) following the manufacturer's instructions. The threshold for positivity defined by the manufacturer was 7.5 U/mL and the range of measurement on undiluted serum was 0.95–4000 U/mL. The PLS was calculated using the formula: exp. [(HbA1c [%] − 5.233) × 1.125 + (OGTT90 [mg/dL] − 107.6) × 0.0195 + (IA‐2A *cat* − 1.27) × 0.662] [3, 4]. For the RBA measures, the previously defined IA‐2A categories used in the formula were < 3 NIDDK units (negative, category 0), > 3–100 NIDDK units, (category 1) > 100–290 NIDDK units (category 2) and > 290 NIDDK units (category 3). For the PLS, three risk groups were previously defined as low (PLS < 0.5), moderate (PLS 0.5–4.0) and high (PLS > 4.0). Progression to stage 3 was assessed for each risk group using the Kaplan–Meier method with follow‐up from the date of the measured PLS until the date of onset of stage 3 or the last contact if the participant had not developed stage 3.

## 3 Results

**FIGURE 1:**
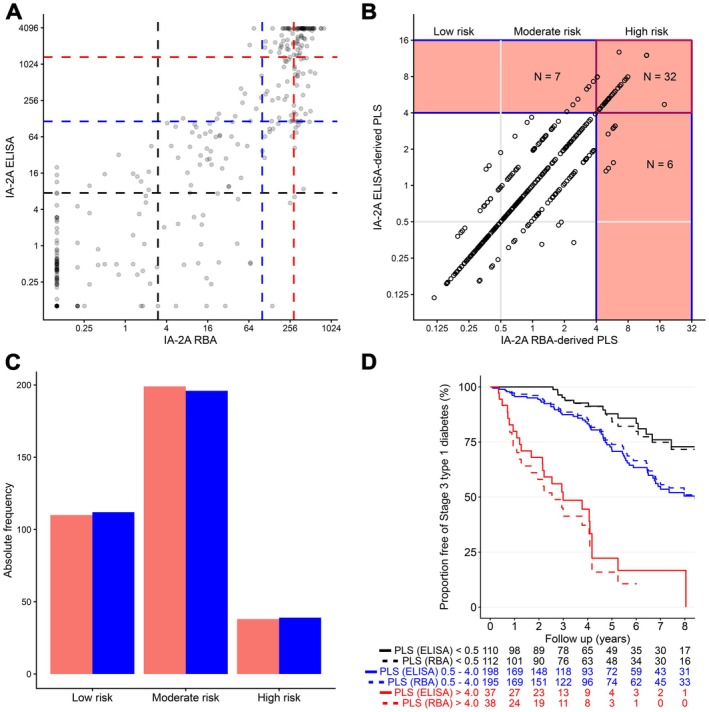
Comparison of IA‐2A measured by ELISA and RBA in children with Stage 1 type 1 diabetes. (A) Comparison of values obtained in the ELISA (*y* axis) and RBA (*x* axis) from 349 children with stage 1. The vertical dashed lines indicate the IA‐2A thresholds in the RBA that define IA‐2A categories used in calculating the progression likelihood score (PLS) and the horizontal dashed lines are the corresponding thresholds calculated for the ELISA. The darker dots indicate more than one data point. (B) Comparison of PLS values derived from the ELISA (*y* axis) and RBA (*x* axis) IA‐2A measurements in 347 children with stage 1. The low, moderate and high risk groups are indicated, which the high risk group (PLS > 4.0) shaded in pink. The number of children with scores in the concordant and discordant high risk groups are indicated. (C) Histogram indicating the number of children in the low, moderate and high risk PLS groups using the RBA IA‐2A measurements (blue) or the ELISA IA‐2A measurements (red). (D) Stratification of progression from stage 1 to stage 3 type 1 diabetes by the PLS calculated using IA‐2A units measured by RBA (dashed lines) and ELISA (solid lines). Children were categorised using previously defined PLS thresholds as < 0.5 (low, black line), 0.5–4.0 (intermediate, blue line) and > 4.0 (high, red line). Progression differed significantly among categories in both the PLS derived from the ELISA IA‐2A units (*p* < 0.0001) and the RBA IA‐2A units (*p* < 0.0001). The numbers underneath the *x* axis indicate the number remaining at each year of follow‐up.

Of the 349 samples measured by both RBA and ELISA, a positive value for IA‐2A was observed in 215 (61.6%) when measured by RBA and in 206 (59.0%) when measured by the ELISA (*p* = 0.54), including 193 (55.3%) positive in both assays (Figure 1). Among those who were IA‐2A positive by RBA, the frequencies in each of the PLS IA‐2A categories were 31% (66/215) for category 1, 26% (57/215) for category 2 and 43% (92/215) for category 3. To align thresholds between the RBA and the ELISA, the corresponding centiles were examined in the ELISA positive samples and corresponded to 7.5–114 units for category 1 (61/205, 30%), 115–1340 (54/206, 26%) for category 2 and > 1340 for category 3 (91/206, 44%). Using these thresholds, a PLS based on IA‐2A ELISA values was calculated for 347 children. Of these, 310 (89%) were assigned to PLS risk groups that were the same as the RBA IA‐2A‐derived PLS groups, 24 (7%) to a higher risk group and 13 (4%) to a lower risk group than those derived from the RBA IA‐2A PLS (Figure 1B). The frequency of children in the low, moderate and high risk groups was similar using the RBA and ELISA IA‐2A derived PLS risk groups (Figure 1C). In particular, the high risk group was assigned to 38 children by the RBA IA‐2A‐derived PLS and 43 by the ELISA IA‐2A‐derived PLS. Furthermore, the ELISA IA‐2A derived PLS risk groups were able to stratify progression to stage 3 in a similar manner to the RBA IA‐2A derived PLS groups (Figure 1D). The 3‐year progression rate in those with an ELISA IA‐2A PLS > 4.0 was 52.4% (95% CI, 30.5–66.1), which was similar to the progression in those with an RBA IA‐2A PLS > 4.0 was 58.7% (95% CI, 37.1–72.8).

## 4 Conclusions

The PLS has practical value for stratifying risk within stage 1. It is derived from measurements readily available from staging, namely, the OGTT, HbA1c and the IA‐2A titre, without additional samples or measurements. The PLS relies on IA‐2A titre categories. Here, we show that although there are some discrepancies between the historical harmonized radiobinding assay and the certified commercial ELISA for IA‐2A measurement, a PLS > 4.0 using either assay in children with stage 1 type 1 diabetes was associated with a faster rate of progression to stage 3 disease approaching the rates seen in children with stage 2 disease [2]. A similar finding was previously reported when the MSD ECL assay was used for IA‐2A measurement [4]. These findings demonstrate that the PLS robustly identifies a subgroup of children with stage 1 disease who are at high risk of rapid progression to stage 3 and support its use to select individuals for clinical trials, irrespective of the IA‐2A assay employed. The ability to calculate the PLS using commercially available assays enhances its accessibility and scalability, allowing broad implementation of the PLS across centres including central laboratories used for eligibility in clinical trials. This, along with its practicality, strengthens the potential of the PLS in risk stratification and trial enrichment, accelerating the development of preventive and therapeutic interventions in early‐stage disease.

## Author Contributions

Design: E.B. and A.Z. Conduct/data collection: M.S. Analysis: E.B. and A.W. Writing manuscript: E.B. and AZ.

## Funding

This study was supported by a grant from the EASD‐Novo Nordisk Foundation Diabetes Prize for Excellence (NNF22SA0081044), and the German Center for Diabetes Research ([DZD] e.V.).

## Conflicts of Interest

A.G.‐Z. served as a member of advisory boards for Sanofi‐Aventis and Novo Nordisk, and received support to give lectures sponsored by Sanofi‐Aventis. E.B. received support for travel and accommodation to attend international conferences and to give lectures sponsored by Sanofi‐Aventis. A.W. and M.S. declare no conflicts of interest.

## Data Availability

All data produced in the present study are available upon reasonable request to the authors.
